# Depression with pain co morbidity effect on quality of life among HIV positive patients in Uganda: a cross sectional study

**DOI:** 10.1186/s12955-015-0403-5

**Published:** 2015-12-30

**Authors:** Emmanuel K. Mwesiga, Levi Mugenyi, Noeline Nakasujja, Shirley Moore, Mark Kaddumukasa, Martha Sajatovic

**Affiliations:** College of Health Sciences, Makerere University, 7072 Kampala, Uganda; Neurological and Behavioral Outcomes Center, University Hospital Case Medical Center, Case Western Reserve University, 11100 Euclid Ave, Cleveland, OH 44106 USA; Infectious Disease Research Collaboration, Mulago Hill Road, MUJHU3 Building, P.O. Box 7475, Kampala, Uganda

**Keywords:** Co morbidity, Pain, Depression, Quality of life

## Abstract

**Background:**

Depression with pain comorbidity (DPC) has not been clearly defined among HIV positive patients in sub-Saharan Africa. It still remains a challenge despite many studies in Africa documenting a high prevalence of pain and depression among people living with HIV/AIDS. Both are associated with a grave impact on the health related outcomes in this pandemic. This study aimed at determining the prevalence, factors associated and effect on quality of life of DPC among HIV positive patients.

**Methods:**

In a cross-sectional survey, 345 HIV positive patients were enrolled into the study. Using a pre-tested standardised questionnaire the presence of DPC was assessed after a written informed consent. The associations between DPC, quality of life, depression history, severity, and cognition were determined. A *p*-value of <0.05 was considered to be significant.

**Results:**

Among people living with HIV/AIDS (PLWHA), the prevalence of DPC was about 5 %. PLWHA with DPC were more likely to perceive their overall quality of life as poor and scored poorly in all the domains on the WHOQOL-BREF. They were also more likely to have more severe forms of depression and recurrent episodes of depression.

**Conclusions:**

DPC is common, under diagnosed and undertreated in PLWHA in Uganda. Depression and pain screening as well as appropriate access to care for DPC have potential to improve quality of life and health outcomes. This calls for the integration and training of mental health services into HIV/AIDS care and future efforts by policy makers and HIV caregivers to address this treatment gap to advance the care of people living with HIV in Uganda.

## Background

Human immune deficiency virus (HIV) disease still remains one of the greatest health challenges in sub-Saharan Africa [1]. It has claimed over 15 million lives, devastated economies, families, and the region as a whole with billions of dollars spent in trying to curb the scourge [1].

With the introduction of Highly Active Antiretroviral Therapy (HAART), people living with HIV/AIDS (PLWHA) are able to live longer and have improved livelihoods [2]. Management of HIV/AIDS, therefore, has been forced to shift towards management of a chronic illness with long term care and different challenges across the spectrum of care. Many factors have been associated with decreased quality of life in PLWHA, but two conditions that seem to stand out in various studies are pain and HIV associated depression [3–6].

HIV-associated depression is more prevalent among PLWHA with depression rates ranging from 8 to 40 % which may be about two times higher than in the normal population [7–10]. It is associated with poorer outcomes especially in terms of adherence [11], substance use and condom use [12] and has been associated with faster disease progression [13]. Pain has been associated with increased drug use, poor quality of life (QOL) and increased morbidity and mortality [6, 14].

Pain and depression have been extensively studied in this population and the two conditions have been found to be highly associated with each other [15–17]. The mesolimbic system together with the neurotransmitters of dopamine and brain indole amine 2,3-dioxygenase have been suggested as being key to the development and interplay of the co morbidity [18–20]. Despite having similar underlying mechanisms, none of the studies however examined depression with pain co morbidity. Studies that have documented the burden of pain in our setting did not assess for depression [14, 21, 22]. Similarly, a number of studies in Uganda have documented the burden of depression in PLWHA without assessing for pain in their participants [12, 22–26]. Some of these studies used tools that assess for acute pain while others assessed for chronic pain [6, 27]. Many studies used screening tools and not diagnostic tools for depression while others looked for psychological distress which is not the same as depression [24, 26]. The concurrent presence of painful conditions and depression contributes to the overall symptom burden among patients. These in turn further exacerbate each other and make it difficult to treat as well as sort out the causal relationships. Therefore determining this co morbidity among our patients would help in managing the affected individuals and improving health outcomes including quality of life.

Despite the prevalence of co-morbid depression and pain among PLWHA, the relative contributions of pain intensity and severity of mood disturbance to overall life quality are rarely examined [9]. Patients with depression and somatic complaints like pain have been shown to have worse quality of life in non HIV populations [28, 29] Studies carried out also in non HIV populations have noted that the quality of life of people with depression with pain co morbidity is worse than those with either condition alone [30, 31]. Few studies have explored the interplay of depression and pain among PLWHA and none in Uganda which has a high burden of HIV. These studies done in our setting have also not assessed the quality of life in PLWHA who have the co morbidity. These are important omissions since a better understanding of pain-depression relationships to quality of life may help guide treatment strategy and help us develop protocols for better patient care.

In this study, we described the point prevalence and associated factors of depression with pain co morbidity (DPC) among HIV positive patients attending a centre of excellence for HIV care in Uganda.

## Methods

### Study design and setting

In a cross sectional study, 345 subjects were enrolled into this study from Mildmay Uganda. Mildmay Uganda is a specialised HIV care centre located at Lweza, 12 km from Kampala the Ugandan capital. Currently, over 10,000 clients (78 % of whom are adults) attend and receive care there. The centre works 4 days a week in an outpatient setting and provides holistic HIV care by employing clinicians, counsellors and social workers. Those requiring specialized care are referred either to physicians at the site or to the National Referral Hospital at Mulago. Adult patients who are too ill are also referred to the national referral for inpatient care.

Senior consultant psychiatrists run an HIV/AIDS mental health clinic once weekly. The centre also provides psychiatric drugs for the patients that needed them as well as counselling and social work services. The centre also does pain assessment at every clinic visit using a numerical rating scale of 1 to 10 and treats patients with pain using clinical assessment.

#### Study procedure

Participants were enrolled if they were receiving care from Mildmay Uganda during the study period, above 18 years of age and consented to participate in the study. Exclusion criteria included those with pain less than 2 weeks or severe cognitive impairment using Mini Mental State examination. Patients on analgesics and anti-depressant medications were not excluded from the assessment. Participants included both HAART and HAART naive individuals.

Trained clinical psychologists obtained written consent from the sampled participants and conducted a cognitive screening assessment followed by a diagnostic interview for depression as well as and an assessment for quality of life. Using the IASP Classification of Chronic Pain [32], a psychiatrist then assessed for pain in participants who reported pain of more than 2 weeks duration. From a review of medical records, recent CD4 cell counts, current HAART regimens and past treatments or diagnosis for pain and HIV associated depression were recorded for all study participants.

**Fig. 1:**
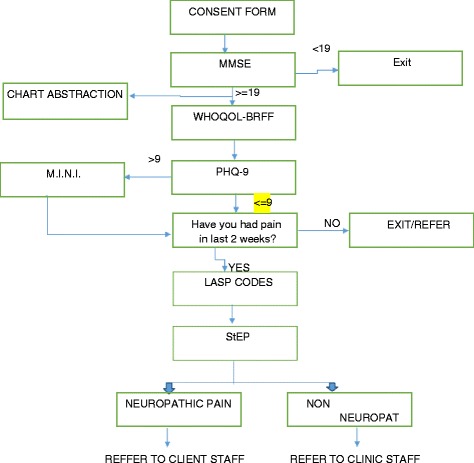
Procedural Flow Chart. (MMSE = Mini Mental Status Examination; WHOQOL-BREF = World Health Organization Quality of Life Brief Version; M.I.N.I. = Mini International Neuropsychiatric Inventory; PHQ-9 = Patient Health Questionnaire- Nine Item; IASP = International Association for the Study of Pain; StEP = Standardized Evaluation of Pain)

See procedural flow chart is shown in Fig. 1.

Data were collected over the 4 month period of September to December 2013. Approvals for the study were obtained from all relevant authorities including the Makerere University College of Health Sciences Ethical Committee (#REC REF 2013–079), Mildmay Uganda Research and Ethics Committee and the Uganda National Council of Science and Technology (HS1455).

Patients found to have pain were referred to the clinic doctor for management of the pain. For patients who scored more than nine on the PHQ and positive diagnosis for depression on the MINI were referred to the specialist psychiatrist present at the Mildmay Centre for treatment while those who scored between four and nine on the PHQ were referred to the counsellors. Patients with quality of life scores below the average were referred to the social work for proper assessment and management.

### Measures

- Socio-demographic information including marital status, age, gender and education level.
- Cognitive scores using the Mini Mental Status Examination. This was used as a screening tool and participants who scored less than 19 out of a maximum score of 30 were excluded due to the fact that patients who score less than 19 have severe cognitive impairment and may not be able to clearly answer all the interview questions. Cognitive scores were then classified according to moderate (19–24) mild (25–27) and normal (28–30) and these categories were used to test for associations.
- Depression severity and diagnosis using the 9-item Patient Health Questionnaire/PhQ-9 [32] and the Mini International Neuropsychiatric Inventory (MINI) [33] respectively both of which have been validated for use in our settings [34]. The PHQ 9 was used as screening and severity assessment tool. Patients who scored more than nine out of a possible scored of 27 on the PHQ had further diagnostic assessment with the section for a Major Depressive Episode from the MINI. A patient who had five or more positive responses on the MINI was diagnosed with a Major Depressive Episode.
- Quality of life assessment using the World Health Organization Quality of Life Scale, Brief Version(WHOQOL- BREF) [35]; that has also been validated for use in our setting. As a preamble the tool collects general information on the patient including whether the patient is feeling ill and if so what specifically if wrong with their health. The tool assesses quality based on four main domains of physical health, psychological, social relations and environment. Higher scores than the mean in a specific domain point to better quality of life. The mean scores and standard deviation for each domain were calculated and unpaired t tests used in testing for associations.
- Chronic pain was described as any participant who had pain of more than 2 weeks duration. Participants who reported in the affirmative then had this Chronic pain classified according to the Classification of Chronic Pain by the International Association for the Study of Pain [36] that describes pain according to the region, system, characteristics, duration and aetiology of the pain.

### Statistical analysis

Using the Leslie Kish formula for sample size determination [37] we took into consideration earlier work which looked for pain in HIV positive populations and also described a sub group of participants who had both pain and depression [38]. Using an anticipated population proportion of *P* = 42.8 %, we needed 357 patients to estimate the prevalence of DPC with 12 % relative precision at 0.05 significance level. However, after cleaning records, a sample of 345 patients was achieved and used. Participants were divided into four different groups of depression alone, pain alone, depression with pain co morbidity (DPC) and those who had no depression or pain. Descriptive statistics of the entire population and the four groups were then presented. Depression with pain co morbidity was described as participants who reported having both pain of more than 2 weeks duration and a diagnosis of a major depressive episode on the MINI. Depression alone was described as those who reported no pain in the previous 2 weeks but had a diagnosis of Major Depressive Episode on the MINI. Pain alone was described as participants who had pain of more than two weeks duration but no depression on the MINI.

In testing for associations the presence of DPC was the dependent variable. Socio-demographic factors, clinical characteristics, mean quality of life scores, depression severity scores, cognitive scores and past depression and treatment diagnoses or treatment were the independent variables. Factors found statistically significant at bi-variable analysis (*p*-value <0.05) were entered into a multiple logistic regression and model building done using a likelihood ratio test (LRT). Co linearity analysis was done among the physical, psychological and environmental quality of life domain predictor variables as well as variables in the multivariable analysis. Those variables with a variance inflation factor (VIF) ≥10 were considered potential causes for multi-collinearity problem. All the domain variables were highly collinear with VIF for physical, psychological, social and environment equal to 29.02, 81.01, 92.41, and 105.66, respectively. Centering and log transformation of these variables did not solve the problem. However, a model containing the physical domain variable (dropping the others) gave a better fit based on the Akaike’s Information Criteria (AIC, smaller is better) and was considered. A multinomial logistic regression was also done to estimate the risk of having pain alone, or depression alone, or depression with pain comorbidity relative to having no pain or depression. This was done using a baseline-category logit model.

A post-hoc power analysis for the final logistic model was conducted to estimate the power achieved in detecting the differences between the factors levels. Data were analyzed using STATA Version 12 (StataCorp. 2011. *Stata Statistical Software: Release 12*. College Station, TX: StataCorp LP).

## Results

### Socio -demographic characteristics’ of the study participants

Between September and December 2013, 345 subjects were enrolled into this study. The socio-demographic and clinical characteristics (See Table 1) revealed that the median age of the study participants was 35 years (IQR: 30–42). The mean scores for cognitive function on the MMSE were 28.0 (SD 2.5). The few patients not on HAART (56/345) were receiving daily Septrin prophylaxis. The mean CD4 count was 537cells/mm^3^ (SD 40.0). There were 94 (27.3 %) participants of the total sample who reported currently feeling ill with the majority of these 74 (78.7 %) having pain related complaints. The mean scores of all the participants in all domains were higher than average as highlighted in Table 2.

**Table 1:** Descriptive statistics for socio-demographic and clinical characteristics

Characteristics		Frequency (*n* = 345)	Percentages %
Age group (years) (*n* = 343)	20 – 30	103	30.0
	31 – 40	139	40.5
	41 – 50	76	22.2
	>50	25	7.3
	Missing Data	2	0.0
Gender	Female	245	71.2
	Male	100	28.8
Education	None	26	7.5
	Primary	146	42.3
	Secondary	140	40.6
	Tertiary	33	9.6
Marital Status	Single	52	15.1
	Married	154	44.6
	Separated	78	22.6
	Divorced	1	0.3
	Widowed	60	17.4
Cognitive Impairment	19–24 Moderate	46	13.3
	25–27 Mild	44	12.8
	28–30 Normal	255	73.9
Complaints (*n* = 94)	Pain Related	74	78.7
	Depressive Related	2	2.1
	Others	18	19.2
CD4 counts (*n* = 332)	<200/mm^3^	58	17.5
	200–349/mm^3^	74	22.3
	350–499/mm^3^	67	20.2
	>500/mm^3^	133	40.1
	Missing	13	0.0
HAART Regimen	AZT/3TC/NVP	96	27.8
	AZT/3TC/EFV	38	11.0
	3TC/TDF/NVP	33	9.6
	3TC/TDF/EFV	108	31.3
	ATV/FTC/TDF/RTV	7	2.0
	Other	7	2.0
	Non HAART	56	16.2

**Table 2:** Mean Quality of Life Scores for all participants (*n* = 345)

Domains WHOQOL-BREF	Participants Score Mean (SD)	Transformed Scores (0–100) Mean (SD)
Physical Health	15.44 (2.20)	61.76 (8.80)
Psychological	16.07 (2.16)	64.28 (8.64)
Social Relationships	13.77 (2.99)	55.08 (11.96)
Environment	14.66 (1.83)	58.64 (7.32)

### Depression with pain co morbidity

**Fig. 2:**
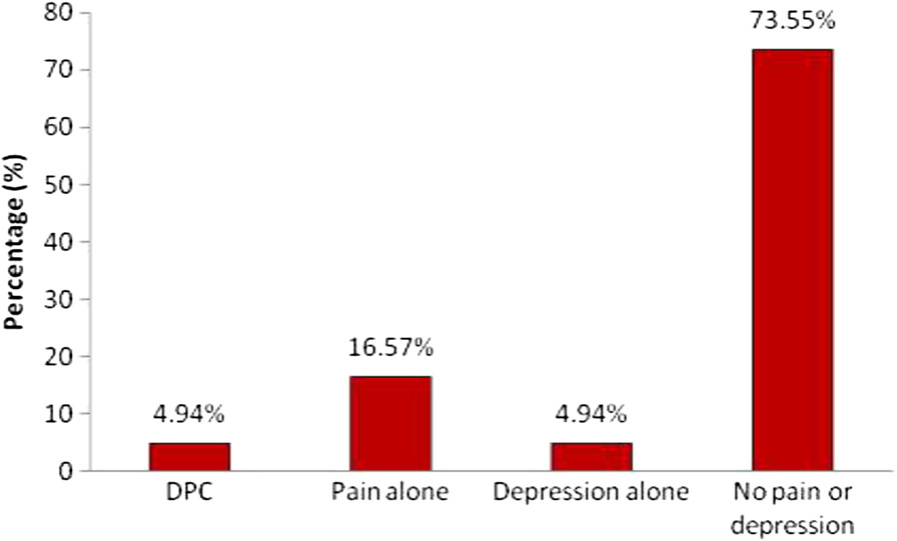
Prevalence of participants among the four groups (*n* = 345). DPC = Depression with pain co morbidity

Nearly 5 % of the study participants had features of HIV associated depression alone while 17.0 % had pain syndromes alone. A total of 17 (4.9 %) participants had DPC. (See Fig. 2).

DPC was more prevalent in participants with CD4 counts less than 500 m^3^ 8(52.4 %) and those on HAART 14(82.4 %). There were similar numbers of participants 5 (29.4 %) with co morbidity who were on HAART combinations of AZT/3TC/NVP and 3TC/TDF/EFV.

DPC was more prevalent in the age groups of 20–30 years (41.2 %), among females (64.7 %), at least primary level of education (58.8 %) and people who were married (41.2 %). There were 82.4 % of participants with DPC on HAART but this was not significant ((*p* = 0.85). There were no associations between participants with DPC in terms of age (*p* = 0.87), gender (*p* = 0.54), education (*p* = 0.26), CD4 counts (*p* = 0.11) and cognitive function (*p* = 0.25). Table 3 highlights variables that were significant at bi variable and multivariable analyses for DPC. There was no problem of collinearity among variables in Table 3 with a highest VIF of 1.18. The results including physical domain plus significant variables as those in Table 3 are shown in Table 4. However, the variable “reported ill” becomes insignificant.

**Table 3:** Logistic regression for the socio-demographic and clinical characteristics with depression with pain co morbidity

Variables		Unadjusted OR	P	Adjusted OR	P
		(95 % CI)		(95 % CI)	
Age		0.97 (0.92 – 1.03)	0.319		
Gender:					
	Female	_	_		
	Male	1.37 (0.49 – 3.80)	0.550		
Reported ill:	Yes	10.20 (3.23 – 32.19)	<0.001	7.29 (1.95 – 27.21)	0.003
Prior depression:					
	Yes	6.08 (1.52 – 24.26)	0.011	13.15 (2.08 – 83.04)	0.006
Prior Pain:	Yes	4.11 (1.07 – 15.77)	0.039	0.78 (0.11 – 5.67)	0.803
Recurrent Depression on MINI:					
	Yes	73.10 (21.64 – 246.94)	<0.001		
Depression Severity:					
	0–14	_	_	_	_
	15–27	57.24 (15.62 – 209.82)	<0.001	36.49 (8.30 –160.39)	<0.001

**Table 4:** Logistic regression for the socio-demographic and clinical characteristics plus the physical domain with depression with pain co morbidity

Variables		Adjusted OR	P
		(95 % CI)	
Reported ill:	Yes	2.46 (0.54 – 11.28)	0.246
Prior depression	Yes	19.77 (2.69 – 145.1)	0.003
Prior Pain:	Yes	0.51 (0.07 – 3.62)	0.505
Depression Severity	0–14	_	_
	15–27	9.26 (1.83 – 46.78)	0.007
Physical health		0.59 (0.44 – 0.78)	<0.001

Participants with DPC reported poorer quality of life in all domains compared to the total sample with the poorest mean scores reported in social relationships 13.7 (SD 11.9) and best scores in psychological well being 16.07 (SD 8.4). At bi variable analysis all domains were associated with DPC although there were no associations with the social domain at multivariable analysis as seen in Table 5.

**Table 5:** Bi-variable and multivariable logistic regression for quality of life domains with depression with pain co morbidity

Domains	Scores	Unadjusted	P	Adjusted^a^	P
	Mean(SD)	OR (95 % CI)		OR (95 % CI)	
Physical	44.08 (10.6)	0.52 (0.42 – 0.64)	<0.001	0.54 (0.41 – 0.70)	<0.001
Psychological	54.12 (11.8)	0.68 (0.57 – 0.80)	<0.001	0.71 (0.58 – 0.86)	<0.001
Social	49.56 (12.04)	0.86 (0.75 – 1.00)	0.054	0.94 (0.79 – 1.13)	0.520
Environment	48.92 (9.44)	0.58 (0.46 – 0.72)	<0.001	0.61 (0.47 – 0.80)	<0.001

^a^Each quality of life domain adjusted for reported illness and prior depression

The statistically significant variables: being ill, a past diagnosis of depression, cognitive function and the four domains of the quality of life were further assessed using a multinomial logistic regression model to determine the relative risk of having DPC, pain alone or depression alone relative to those with neither pain nor depression and the results are shown in the Table 6 below.

**Table 6:** Adjusted estimated risk ratios (RR) for factors associated with pain, depression, comorbidity and non-comorbidity among participants using multinomial logistic regression

	HIV Pain alone		HIV associated depression alone		Depression with pain co morbidity	
Adjusted effects	Adjusted RR (95 % CI)	P	Adjusted RR (95 % CI)	P	Adjusted RR (95 % CI)	P
Cognitive Function:						
Moderate	_	_	_	_	_	_
Mild	0.71(0.19, 0.58)	0.60	0.35 (0.06, 0.90)	0.22	0.67 (0.07, 6.71)	0.74
Normal	0.94 (0.37, 2.41)	0.90	0.22 (0.06, 0.77)	0.02	0.58 (0.11, 3.05)	0.52
Reportedly ill:						
No	_	_	_	_	_	_
Yes	7.70 (3.82, 15.51)	0.00	1.64 (0.48, 5.64)	0.43	5.39 (1.18, 24.65)	0.03
Past Depression Treatment:						
No	_	_	_	_	_	_
Yes	0.57 (0.06, 5.42)	0.62	0.00	0.99	11.39 (1.33, 97.14)	0.03
Physical Health						
	0.66 (0.55, 0.79)	0.00	0.51 (0.40, 0.66)	0.00	0.39 (0.28, 0.52)	0.00

## Discussion

Despite the fact that many studies have looked at depression and pain in our setting, this is the first study in Uganda to specifically assess DPC as a distinct syndrome among individuals with HIV. Few studies in Africa have explored this co morbidity among HIV positive populations. Our study found a prevalence of 4.9 % is much lower than studies by Miaskowski et al. [39] who reported a prevalence of 42.8 % in a community sample in the United States and Rosenfield et al. [40] who reported a prevalence of 10.5 % in a hospital setting in the United States. The difference in prevalence in our setting might be explained by the integration of specialised services such as mental health care provision that may provide better ways to manage DPC. Also lower prevalence of HIV as well as the difference between community, hospital and outpatient settings might have affected outcomes.

DPC was associated with more severe depression, recurrent /previous depression, and lower quality of life in three of the four domains assessed. These results are similar to other studies that highlight the impact of DPC on quality of life in PLWHA [4, 6, 22, 41, 42]. Our study contrasts the relationship in quality of life between participants with pain alone, depression alone and with DPC. Participants with DPC had worse quality of life than participants with HIV- associated depression alone and HIV pain alone. These results remained significant even after adjusting for other factors. There is therefore need to screen for and mange depression with pain co morbidity as our findings suggest that it has worse impact on quality of life than either depression or pain alone.

In our sample, past pain diagnoses and treatment were not associated with DPC. A past diagnosis of depression was however associated with the co morbidity. Past depression may therefore be a better predictor for DPC. This differs from studies by Tsao et al. that suggested pain as a mediator for depression in PLWHA [8]. In our study we also found that depression severity and not pain intensity was associated with DPC even after adjusting for other factors. These results mirror a study by Keltner et al. who noted that depression severity more than pain intensity was associated more with HIV associated distal sensory polyneuropathy [3]. This implies depression severity moderates both pain and DPC. The role of depression history, presentation severity and treatment in the presentation of DPC in PLWHA will therefore need further analysis.

Earlier studies looked at pain and depression separately in our setting [21, 43] and studies looking at DPC have been mainly in the developed world [38–40]. Looking at pain and depression under separate conditions may underestimate burden and may not be ideal to inform real-world provision of care [44]. This is well highlighted by the fact that about half of the participants who had moderate to severe depression on the PHQ-9 and all the participants who had depression alone on the MINI had some form of pain complaints. Assessing for pain in patients with depression in PLWHA is therefore imperative.

Also, the risk of having HIV associated depression alone for patients who reported feeling ill was smaller compared to being co morbid or having pain alone. Feeling ill was thus more associated with HIV pain alone than DPC or HIV associated depression alone. This drop may point to the fact that many of the people who had depressive symptoms also had pain or were reporting depression as pain and feeling ill may thus be a culturally appropriate way to describe pain in the Uganda setting. These findings are similar to a study by Okello et al. [45] on the role of somatisation of emotional problems in Uganda. Further studies are needed on whether pain may actually be a symptom for depression as the study design cannot determine causality. This might actually lead to better screening tools that consider the cultural context of somatisation of emotional disorders in our setting as noted by Okello et al. [45].

Screening as well as early and appropriate management of DPC in PLWHA may help improve quality of life. Simple and self-rated instruments such as those used in this study could easily be implemented into routine practice settings to get patients with DPC into care that is appropriate for their situation. The IASP classification of chronic pain, though not frequently used in HIV populations; together with the PHQ-9 and MINI are easy to administer tools that might be able to readily identify people with DPC among PLWHA in sub Saharan Africa. Due to chronic shortage of staff in sub Saharan Africa, these screens are thus ideal in routine clinical care as they are self-rated and do not require a lot of healthcare or personnel resources.

## Conclusions

One in 20 persons was found to have DPC and this was associated with greater depression severity, past depression diagnoses and worse quality of life. DPC appears to be a distinct syndrome that must be actively screened for and treated. It is important for palliative care physicians who are trying to develop integrated palliative care models to care [44] to be aware of DPC and the factors associated with it. Efforts to diagnose and treat HIV associated depression may prevent future pain and depression with pain co morbidity as well as having possible positive impact on quality of life. It is therefore important for psychiatrists who frequently have to deal with somatic complaints and the somatisation of emotional illness. It is also important for palliative care physicians who are trying to develop integrated palliative care models to care [44] to be aware of DPC and the factors associated with it.

Pre-emptive depression and pain screening in people with HIV, followed up by focused referral and treatment could advance the care of people with HIV that is complicated by DPC. This calls on doctors in Uganda to look for non physical causes of pain, especially depression; in patients with HIV/AIDS.

### Limitations

The cross sectional nature of this study makes it difficult to examine causation. More studies are required in order to better understand the associations and impact of the factors examined above on patient outcomes. The fact that patients on treatment for pain and depression were not excluded may also be a limitation as we could not clearly define the severity of the ailments. There is also a slight possibility of measurement bias for pain as in our setting many pain medications are over the counter medications. These patients on treatment for pain and depression were few but highlighted the under treatment and under diagnosis of these two conditions in PLWHA. Also, the low number of outcome events per predictor variable in our multivariable models actually limits the robustness of our results. Further research is needed to confirm our findings, which shall be interpreted as descriptive so far.

## Abbreviations

DPC: depression with pain co morbidity
HAART: highly active antiretroviral therapy
HIV: human immunodeficiency virus
MINI: mini international neuropsychiatric inventory
MMSE: mini mental status examination
PLWHA: people living with HIV and AIDS
